# Confessions of a baboon watcher: from inside to outside the paradigm

**DOI:** 10.1007/s10329-023-01060-1

**Published:** 2023-05-10

**Authors:** Shirley C. Strum

**Affiliations:** The Graduate Division, School of Social Sciences, Department of Anthropology, University of California, San Diego, 9500 Gilman Drive, La Jolla, San Diego, CA 92093-0532 USA

**Keywords:** Olive baboons, Social strategies, Primate mind, Community-based conservation, Evolution, Natural history

## Abstract

In this “tale” I summarize the major landmarks of my 50-year career watching wild olive baboons (*Papio anubis*). I review some major discoveries, like baboon hunting and baboon social strategies of competition and defense, that only a creature with a “mind” could manage. My efforts expanded beyond science to include community-based conservation because quite early on these baboons experienced many of the threats of the Anthropocene. My research expanded to include studying crop-raiding by naïve groups of baboons, the first scientific translocation of a primate species, and a detour to study the invasion of a non-indigenous cactus, *Opuntia stricta*. Throughout I worked with local communities to find solutions to problems that the baboons created, and also to develop new options for their livelihoods. As the baboon research became a long-term project, it depended on a team of Kenyan research assistants who made possible the simultaneous monitoring of up to six baboon troops as well as extensive ecological monitoring. Knowing the ecology, including the impact of the sedentarization of pastoralists in the area, meant we could interpret the process of invasion by a non-indigenous cactus for the first time. Ecological periods allowed comparisons of the same troop over time and different baboon groups during the same ecological phase. Although I began my work before hypothesis testing was the preferred approach, once the paradigm changed, I continued to study and learn what matters to baboons from their perspective. As a result of observing them for 50 years, the baboons showed me that evolution often does not work the way that I had been taught, and it took all my detours and studies to convince me that anecdotes, when they are systematic and comparative, are not stories to be discounted, but evidence, much like Darwin’s natural history. Natural history can reassemble the pieces that quantitative hypothesis testing has teased apart to provide its larger meaning. Today, the lone scientist, like me, is an anachronism because no one person has expertise in the many fields needed to understand and save the primates we care about.

## Starting out (1965–1972)

No one would have guessed when I was a freshman at the University of California Berkeley in 1965 where I would end up. Looking back, I see that I have been the beneficiary of circumstances, not its victim.

As an undergraduate at Berkeley, I looked for a scientific way to study human behavior. I toyed with sociology, psychology, even criminology, but my first physical anthropology class with Sherwood Washburn hooked me on the evolutionary perspective. Washburn’s charisma mesmerized all 1000 of us students. You could hear a pin drop. He had defined “the new physical anthropology” (Washburn 1951a) for American anthropologists by injecting the “new synthesis” of genetics from systematics and paleontology (Mayr 1942; Huxley 1974) into physical anthropology. He also argued that scientists should watch how primates move and behave in their natural environment to understand anatomy, fossils, and human evolution (Washburn 1951b), and thus launched a major wave of primate studies in the 1950s and 1960s (Washburn 1951a). From today’s perspective, it is surprising how little we knew in the 1960s and early 1970s about nonhuman primates, particularly species living in the wild (Strum and Fedigan 2000a). Back then, the “good of the group” interpretations were acceptable and scientists still puzzled over how to trade off phylogenetic relatedness and ecological context (Gartlan 1968; Struhsaker 1969). Even social carnivores were considered possible models for human evolution (Schaller and Lowther 1969).

**Fig. 1:**
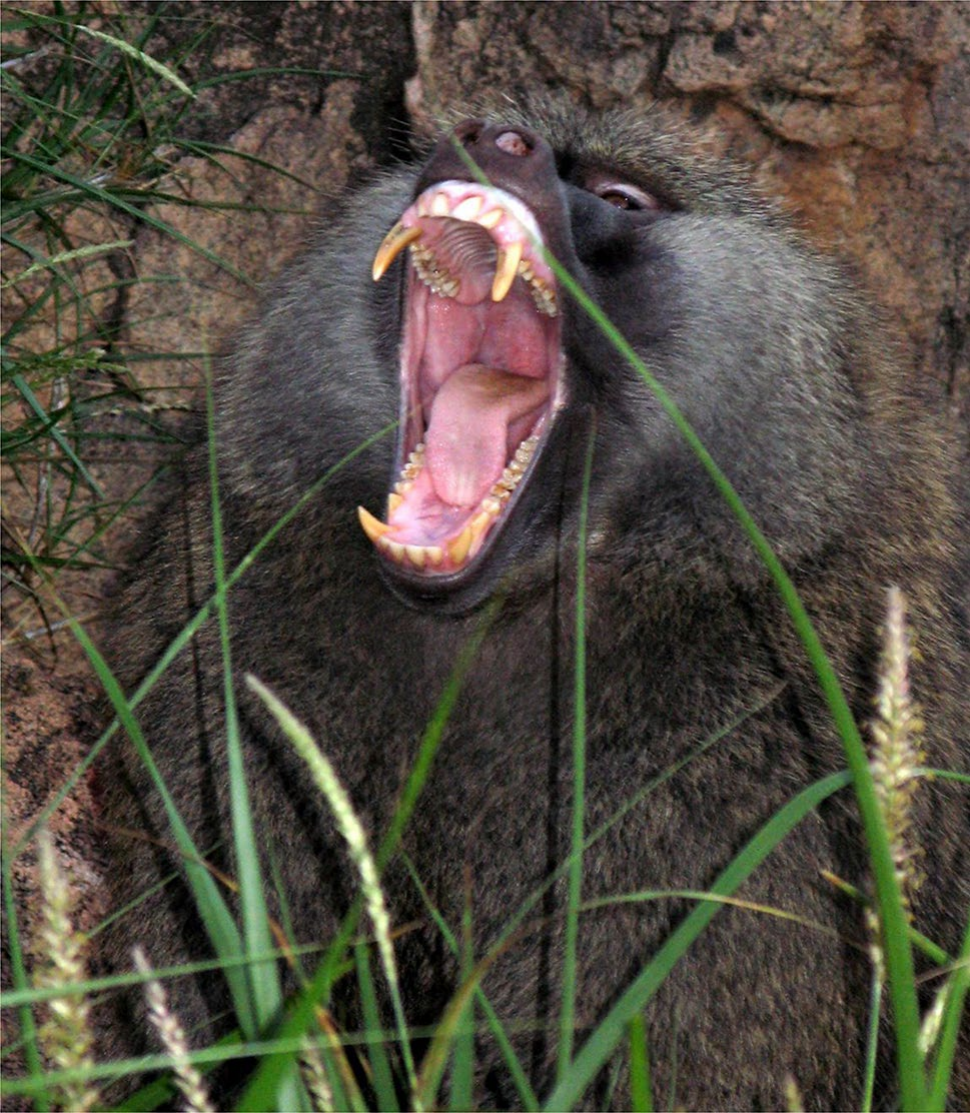
The baboon model and the anatomy of aggression

Washburn became my mentor as an undergraduate and then in graduate school at Berkeley. Among Washburn’s many accomplishments, he helped create the “baboon model” of primate behavior based on evidence from field studies by his student Irven DeVore who watched olive baboons (*Papio anubis*) in Nairobi National Park in Kenya in 1958 (DeVore and Hall 1965), his own study of yellow baboons (*Papio cynocephalus*) in the Amboseli ecosystem in Kenya in 1959 (Washburn and DeVore 1961), and K.R.L. Hall’s study of chacma baboons (*Papio doguera*) in Southern Africa in 1958 (Hall 1965). The baboon model emerged as the scientists compared results (Washburn and DeVore 1961; DeVore and Washburn 1963) because the similarities were striking given that the baboons were different species and lived far apart geographically. They concluded that baboons everywhere lived in cohesive societies, staying together throughout the day for feeding, traveling, socializing and resting, and sleeping together in trees or on rocks for greater protection from nighttime predators. The baboon group was large by primate standards and included individuals of all ages and both sexes. Mature females outnumbered mature males, with the majority of the group composed of immatures. Large males moved between groups, a good way to avoid inbreeding, while females stayed in their natal group. The most exciting finding at the time was that male anatomy and behavior seemed to have coevolved for life on the savanna. Adult males were almost twice the size of adult females. They had imposing canines that sharpened against their lower premolars when they opened and closed their mouths and a large mantle of hair around the head and shoulders which made them look even bigger when erect. Here was a rare example of evolution in action as male baboons were equipped with the anatomy of aggression, an adaptation to a new range of large savanna predators that was also useful during male competition for limited resources like food or receptive females (Fig. 1)

.

The baboon model was male-centered, which was not surprising since only the large males were identified as individuals. According to this model, females were not aggressive and apparently did not have a hierarchy or much structure to their relationships with each other. Their role was to nurture the next generation, an important and time-consuming job. Without a female hierarchy, the status of a female depended on her sexual receptivity, so a female could temporarily be high ranking when she was with a high-ranking male, but no longer of that rank when she was on her own. The male dominance hierarchy provided the troop’s structure, and males controlled group politics, protected females and young, and policed internal disputes. Nowhere was the baboon model more apparent than when the group moved through the open savanna. Images of this baboon formation graced the covers of many anthropology and biology textbooks. Dominant males were in the center of the troop, protecting mothers with infants, surrounded by other animals, while low-ranking males were on the edge and could be sacrificed to lions, for example, if the troop was unaware of their presence. The popularity of this model spanned scientific and public audiences because baboon society, and particularly baboon males, showed how evolution and natural selection worked to link behavior and anatomy with environment, which made for a compelling story.

Initially, I wanted to study patas monkeys (*Erythrocebus patas*) because they also lived away from the safety of forest trees but had a very different social organization to baboons (Gartlan 1975). However, Washburn insisted that I study baboons, the most studied primate species at that time. Reluctantly, I went into the field to test the baboon model because baboons had come to represent all primates (Strum and Fedigan 2000a). I wanted to know if this was really correct. Of course, there was already some evidence to the contrary, but at that time it was mostly ignored (Sugiyama 1965; Rowell 1966; Gartlan 1975).

Neither Washburn nor I expected that my findings would so profoundly contradict the picture that he had helped to create. However, when I returned from the field to Berkeley, my newly minted quantitative data got jammed up in the Berkeley computer, which at the time occupied eight floors plus the basement of the building next to Washburn’s office. Given the uncertainty of when I might get the results, Washburn recommended that I write my dissertation on a topic of great interest to anthropologists and a key issue in human evolution: primate predation and hunting. I had collected data on predation as extras during my field study (later I recognized that this was natural history—see below), and I could manually analyze these. However, that meant that, although I had gone into the field to study the value of males and females to baboon society, in the end I published on a tangential topic: baboon predation.

## Baboon predation: a tradition or not? (1972–1976)

**Fig. 2:**
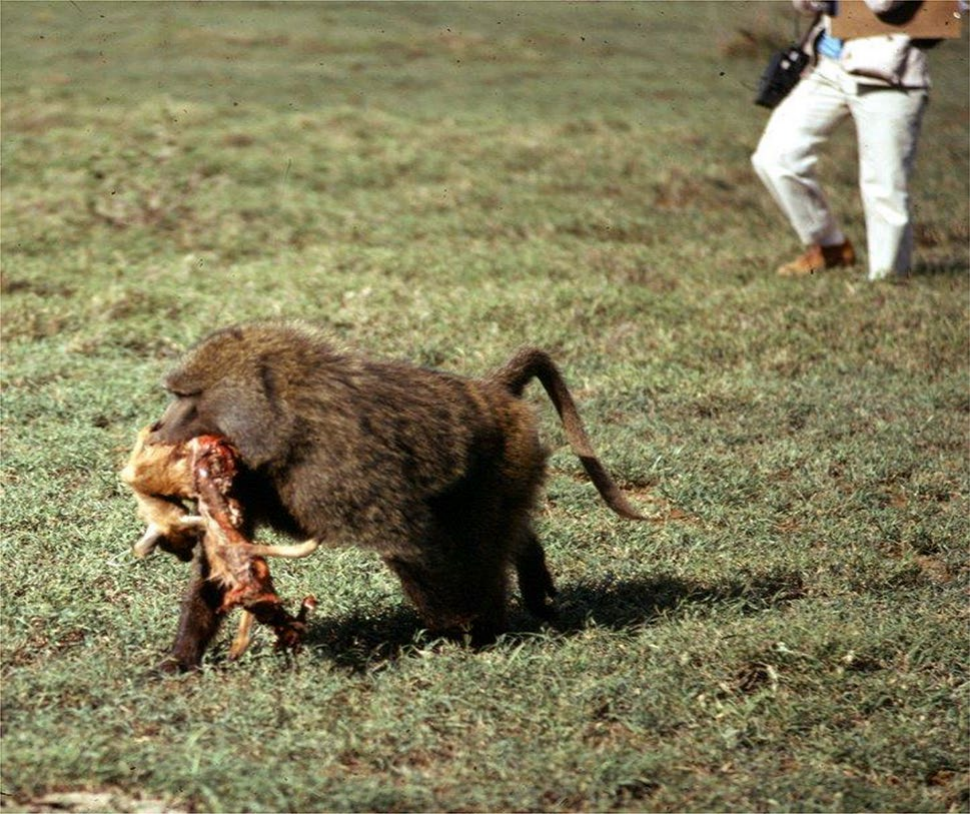
Baboon predation on a Thomson’s gazelle

Man the hunter was a powerful theme in evolutionary scenarios in the 1970s. My data suggested that the baboons that I studied were the most predatory nonhuman primates known at the time (100 episodes of predation in 1000 h of observation), indeed even more predatory than the Gombe chimpanzees (Teleki 1973). I also thought I had observed the development of a hunting tradition, as the males followed each other out of the group and learned to do relay chases towards each other rather than in random directions. This increased their success in capturing Thomson’s gazelles (Strum 1975a, 1981). I wanted to make a distinction between what baboons do and what human hunters do, so I called the baboon behavior “p hunting” and the human behavior “q hunting” just to eliminate all the cognitive baggage associated with the word “hunting.” Reviewers rejected this idea, but the paper (Strum 1975a) was published when my revision removed the terms p and q hunting. By early 1975, baboon hunting was no longer observed, perhaps because the male instigator of this behavior had transferred to another troop. Thus, he did not take the act of hunting with him, or leave it behind. Without census counts of Thomson’s gazelles, I could not know their exact numbers, but the baboons seemed to encounter them at about the same rate as previously. It was only later, by comparing baboon hunting to crop-raiding, that I could speculate about why the hunting tradition did not persist. This shows the value of long-term studies, as through them it is possible to compare events distant in time, including things that were not initially a focus of the research (Fig. 2)

.

## What are male baboons? Social strategies of competition and defense, residency, and hierarchy (1972–1978)

**Fig. 3:**
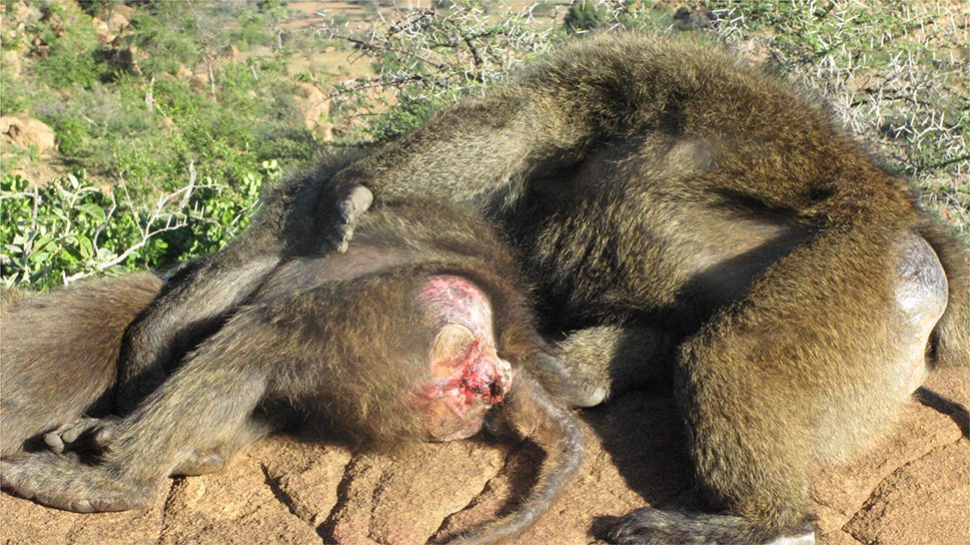
Friendships between males and females are part of social strategies of competition and defense

At the end of my first field stint trying to understand male and female roles in a baboon group, I knew that the baboon model was wrong on several important points. First, females had a stable dominance hierarchy based on families. I already suspected this given the work on rhesus macaques from Cayo Santiago (Sade 1967). Glenn Hausfater also confirmed the female hierarchy in his study of yellow baboons (*P. cynocephalus)* in Amboseli (Hausfater 1975), which overlapped in time with my research. Second, males were very different from the baboon model. My data showed that male baboons lacked a stable dominance hierarchy because rank was short-lived, aggression risky, and the dominance system dynamic. In addition, I had evidence that baboon males not only had the anatomy of aggression but also had non-aggressive, social strategies of competition and defense that relied on establishing and maintaining social relationships between males and females and males and infants (Strum 1975b). Tim Ransom first observed these “special relationships” at Gombe Stream Reserve during his 1967 study (Ransom 1981) but he did not quite see their overall value. Perhaps, in a longer term study, data could document how these unfolded and functioned. The data showed that “residency” among these males, whether they were newcomers, short-term residents or long-term residents, was a better predictor of dominance rank and consort success (Strum 1982) because social strategies took time to develop. Opening my eyes to the value of social relationships for baboons meant thinking about the individuals and their social realm in a different way (Strum 1979). Baboons needed social skills, social intelligence, and social sophistication to manage their social web (Strum 1979, 1983, 1984). When Barbara Smuts came to study female baboons, I insisted she could not understand females without also studying males. The result was one of the first publications that used the term “friendship” instead of special relationship for baboons (Smuts 1985). Females, not just males, also had social strategies, which buffered their smaller size and dominance rank. I began to think of baboons as being both smart and nice because they needed each other to succeed socially (Strum 1987/2001) (Fig. 3)

.

**Fig. 4:**
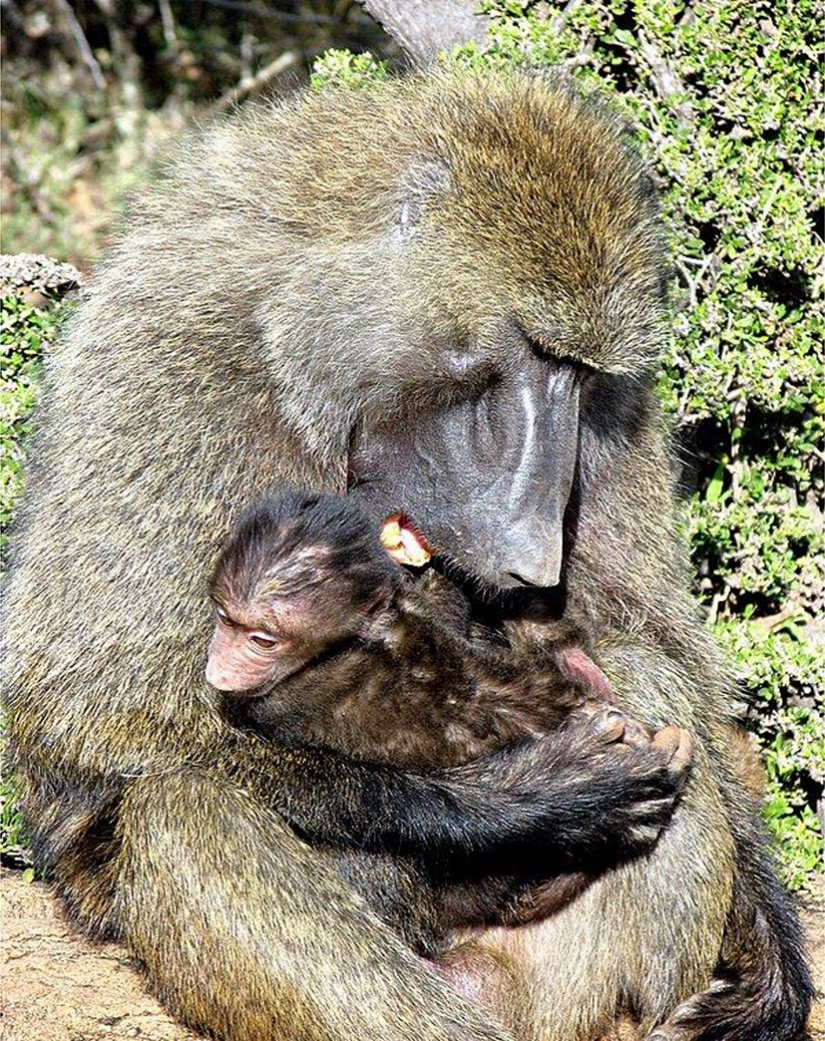
Males use infants as agonistic buffers as well as passports

**Fig. 5:**
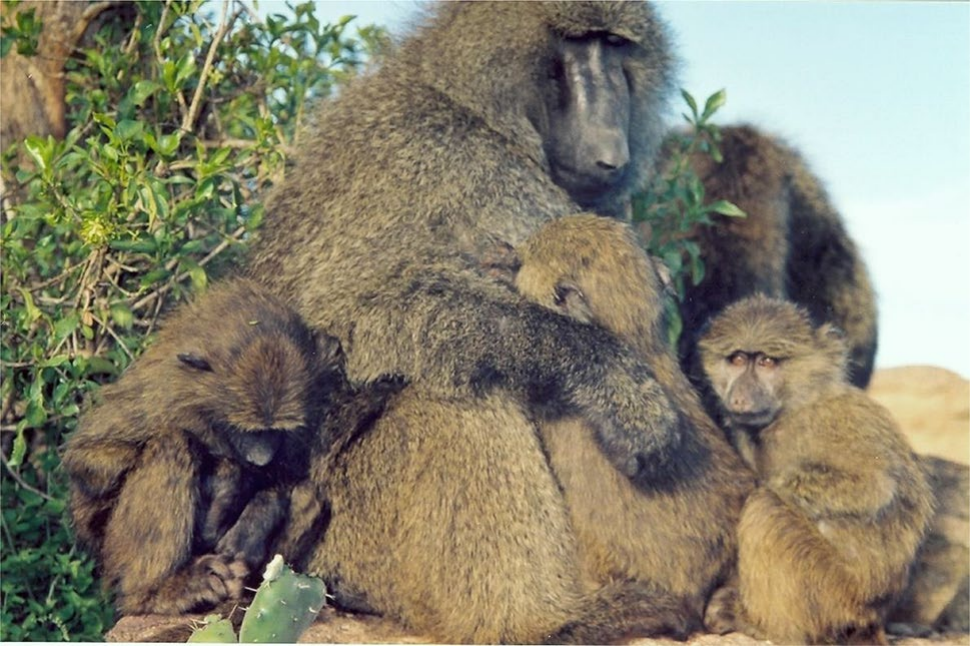
Male–infant friendships last longer than male–female friendships

However, I had problems when I tried to publish my paper on male dominance rank. The reviewers insisted I construct a hierarchy from what I claimed was dynamic. The only criterion that produced a dominance hierarchy was if I counted a male who won 51% of encounters as dominant to his opponent. With this male hierarchy in place, I went on to show that male dominance rank did not correlate with access to two important resources: females and meat from predation by baboons (Strum 1982). Furthermore, when a male was about to lose an aggressive encounter with another male, the potential loser could use an infant or a female as an agonistic buffer (Strum 1983, 1984). Females also had choice in consorts, reflecting special relationships with males, a term I continued to use until I was convinced that baboons, not just humans, had friendships. These relationships might not dictate which male was the sexual consort, but could determine if that male kept the female or even had any copulations (Strum 1987/2001). In these ways, males who were not in their aggressive prime could still get sexual consorts or other desired resources by using their wits (Western and Strum 1983; Strum 1989, 1994a) (Figs. 4 and 5)

.

To use these non-aggressive social strategies, baboons must have a “mind.” In this way, Jane Goodall’s work on chimpanzees and my early baboon research, along with the ape language experiments, helped shift scientific ideas towards acknowledging a “primate mind” (Strum and Fedigan 2000a). Similarly, cognitive ethology (Griffin 1976) and cognitive science, which emerged slowly, changed the focus from behaviorism to one that considered mind in both animals and humans. From this early start, primate studies have been elaborating what it means to have a nonhuman primate, but not a human, mind (Strum 1987/2001; Strum et al. 1997; Strum and Forster 2001).

## Negotiation: social complexity not social complication (1978–1987)

About now, I stopped thinking of baboons as a “model” for human evolution and became fascinated by the baboons themselves. They seemed “almost human” (Strum 1987/2001) to me in their extensive negotiations and social strategies that approximated “politics.” An early collaboration with Bruno Latour helped me to figure out why. Latour and I worked closely for decades; he joined several Wenner-Gren international symposia that I convened, while I took part in several international events that he organized.

We published two papers together. The first was a critical look at how we put together speculative scenarios about human evolution (Latour and Strum 1986), which suggested that we needed new and better “stories.” The second had a big impact on my thinking about baboons which would reach fruition much later (Strum and Latour 1987). We compared baboons to the scientists that Latour studied at the Salk Institute (Latour and Woolgar 1979) and concluded that the two used the same processes to build society because society is not something that individuals enter but something that they create. Yet there were obvious differences between baboon and human society. We suggested that this was the result of the different resources that each species brought to the process of building a society. Baboon society demonstrated social complexity, where everything apart from what is inscribed in the body must be dealt with simultaneously. Humans use their tools, technology, symbols and language to create a society that is socially complicated, like, for example, a computer network or the structure of a snowflake, but one in which, ironically, humans lose social skills because much is simply “black-boxed” (Strum and Latour 1987; Langlitz and Strum 2017).

## A naive group of baboons become crop raiders: abnormal or not? (1981–1984)

The baboons faced what later became major conservation issues. The first example of this was when the land on which the baboons ranged, Kekopey, a cattle ranch with fewer cattle than wildlife, was sold in 1977 to an agricultural cooperative. The pressures of population growth and land use change meant that traditional plots of land had been subdivided between many sons for several generations. Now there was not enough land for each of them to support a single family. People who could, went looking for other possibilities. But Kekopey, although a great place for cattle and wildlife, was not appropriate for rain-fed agriculture. That was not taken into account, though, in people’s desperation to own a piece of land. Understandably, the baboons thought these attempts at growing crops provided great new foods in their traditional home range. The main study group split into raiders and non-raiders (Strum 1987/2001, 2005). The raiders were primarily large subadult males with their female friends. Older sisters left their families in the main group to join raider male friends, while the lowest ranking female, who was not friends with any of the raider males, took 6 months to make up her mind. When she did, her weaned brown infant returned to his brothers in the non-raider group, probably because there were no immatures with whom to play in the raider troop. This was an amazing opportunity to watch how decisions as critical as “which foraging strategy?” get decided. I used evolutionary principles to interpret parts of the baboons’ decisions, but this fission added evidence that baboons have options—not all adolescent males of the right age became raiders, not all older sisters deserted their families, and the lowest ranking female used her own playbook to decide which troop to join.

Raiders, perhaps because they encountered the costs of raiding more often, adjusted to and avoided most of these costs, while the non-raiders paid the highest price in terms of injury and death. Above all, the data showed that raiding was not “aberrant,” as scientists thought at the time; it was a good foraging strategy whenever the benefits outweighed the costs. Although I translocated the baboons before I could document all the benefits of raiding, weight-at-age was higher for the raiders, human-caused deaths declined among raiders, and female raiders had shorter inter-birth intervals after translocation (Strum 1991, 1994b, 2010).

The crop-raiding period let me test both traditional and non-traditional methods of deterrence. For example, we found that it was critical to use up time for the raiders by guarding farms because baboons could wait the whole day for just a short chance to feed on crops. We also tried conditioned taste aversion (Forthman-Quick 1986) and other non-traditional methods, like thunder flashers, leopard dung, and playing baboon alarm vocalizations. In the end, it was traditional guarding of farms or the farming area that worked best.

**Fig. 6:**
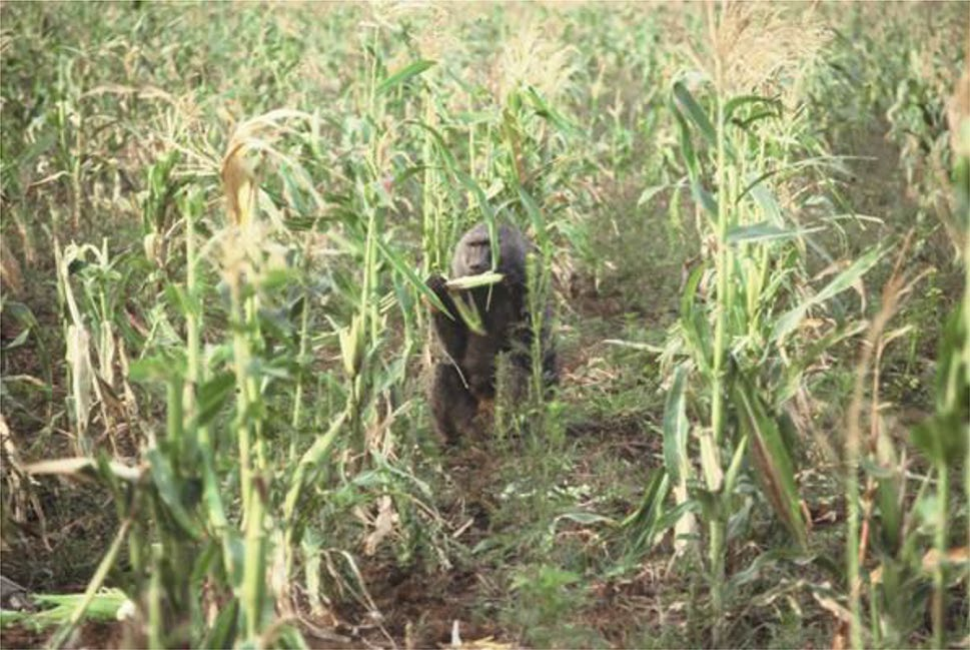
Crop-raiding baboon in a maize field

Crop-raiding became embedded in one of the three baboon troops, unlike hunting. The difference seemed to be, first, that the behavior must happen often (which baboon predation did not) and second, that it must happen in full view of the group (which baboon hunting did not). Baboons do not lack innovations, but social constraints like these prevent an innovation like “hunting” from becoming a “tradition” (Fig. 6).

## Translocation as a management and conservation tool (1984–2000)

**Fig. 7:**
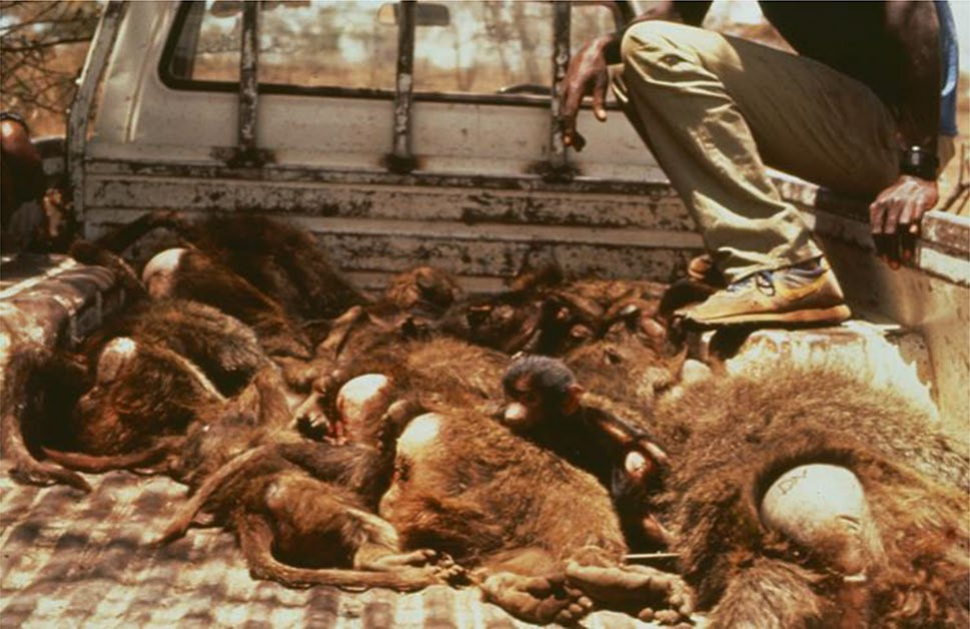
Translocation: transporting baboons from traps to holding cages

The crop-raiding led to conflict not only with would-be farmers but also with a nearby army base, where people had guns. At first, the women in the married housing at the army camp found the baboons entertaining, but then the baboons began to raid their kitchens and conflict escalated. There were only two options: either I leave the baboons there and watch them be selectively killed, or I move them. Once again, this presaged what was to become a conservation tool: translocation. I had more than a decade of information on these baboons which made them scientifically valuable, but equally important was that I wanted to keep them safe. I felt I owed them that in exchange for what they had given me over the past decade. It would also be valuable to test whether translocating a generalist primate succeeded. If not, translocation could not be a good tool for more specialized primate species. But if the baboons did survive translocation, the basics of moving them could then be adapted to the special needs of other primate species. In the early 1980s only few species had been translocated and no primate species had been moved from one place to another in their historical range. But success meant circumventing professional trappers' methods who procured baboons for medical research. Without precedents to follow, I had to invent protocols for all aspects of the move. In August 1984 we began with the troop I deemed least scientifically valuable because it had only been studied for 3 years. The team, including trappers from the Institute of Primate Research, Kenya, completed three translocations, ending with the most scientifically valuable group, the Pumphouse Gang. This order offered the opportunity to learn more about how best to translocate baboons (Strum 2002, 2005). Fortunately, the translocation was a success as measured by several criteria. First, there was a significant shift in sources of mortality before and after translocation, from high human-induced mortality before translocation to natural mortality afterwards. We could also compare translocated troops to an indigenous group under study whose home range overlapped with those of two of the translocated troops. In all measures, including rate of growth in group size, birth rate, infant and adult female survivorship, and mortality rate, the two translocated study groups performed as well as the indigenous group (Strum 2005). For me, the most surprising outcome of the translocation was how much success relied on “social” resources and not on competition with each other over scarce food because 1984 was the worst drought in 20 years (Strum 1987/2001, 2012). The baboons were moved as intact groups so they could rely on each other for support. Unlike captive animals returned to the wild, these baboons already knew how to learn and seemed to know what they needed to learn to survive. The post-translocation monitoring now extends to 38 years and includes matrilines whose history can be traced back to 1972. Translocation has since become a widely used and successful conservation and management tool for primates in the wild (Fig. 7)

.

## Fusions, fissions, confusion, and resolution (2000–2010)

This baboon project evolved to become a long-term research venture, as did many projects that began in the 1970s and afterwards. The fact that it was a long-term project allowed us to track both natural variations (wet seasons, dry seasons, and droughts) and unnatural experiments like the incursion of agriculture, translocation, and the pre- and post-*Opuntia* periods. Comparisons between distinct ecological periods helped us to identify how the social and ecological interacted and what was most important.

Although I identified baboon social complexity in my first study, the baboons offered more evidence of what this meant in the remaining decades. In 1981, I watched the first fission of the study group into raiders and non-raiders. It was another domain in which baboons had options showing that decisions were not pre-determined. I documented other fissions, three of which occurred in 2011, 2012 and 2017 that revealed how baboons make trade-offs about living in a social group. Each fission took upwards of 2 years to complete. These later fissions were the result of the invasion of *Opuntia stricta,* internal tensions between males and females in the mother troop, and the abundance of the sleeping sites. Small male-focused subgroups are normal within a baboon troop, but in these fissions, subgroups split from the mother group when they were very small, and perhaps not even initially viable. Two factors played a central role in two of the fissions: food competition and protection from nighttime predators. However, these factors did not explain the process, only the outcomes. At first, the invading cactus was restricted to one area. There, larger baboon groups had an advantage in gaining access to the cactus plants, as theory predicts. But within a decade the cactus spread widely, so that group size was less important in inter-group competition. This meant smaller groups could access *O. stricta* too. The splinter baboon groups solved the risk of nighttime predation in a novel way. Small daughter groups slept near each other or near the mother troop, so these very small groups were always within earshot of other baboons and their warnings of danger. This  meant that ther small troop baboons no longer had to be members of a big group.  They had the added advantage that when foraging began each day there was less social complexity to monitor (Strum 2012). Up to a point, these fissions can be interpreted as an evolutionary response to feeding competition and predator avoidance, but documenting the process illustrated the baboons’ deliberations and innovations. At no point was the outcome pre-determined. For example, in two of the fissions, both males and females moved between groups over several years, following friends and family. In contrast to predictions, families split between groups and one low-ranking female actually joined the members of an indigenous troop that included none of her relatives. In this way both splits violated baboon rules for troop splintering. One fission also involved a revolution in the female dominance hierarchy, where the top-ranking female and her daughter were evicted from the mother troop and forced to stay in one of the daughter groups. The top-ranking female snuck back into the mother troop but fell in rank.

I also saw fusions of baboon groups at a surprising rate for terrestrial monkeys who do not have a fission/fusion type of social organization. Examining the social and ecological contexts suggested different evolutionary interpretations for each fusion but, once again, paying attention to process modified the interpretation of evolutionary principles. The first fusion happened 17 years after translocation and involved the previous raider troop (now no longer “raiders”) and an indigenous group. The translocated raider troop had declined in size to 13 individuals because of heavy predation. The indigenous group had also rapidly declined from over 70 individuals to just 35 animals because of predation. After the fusion, the troop was once again large and predators avoided it while continuing to prey on a small local troop whose home range overlapped with that of the fused group. This suggested that this fusion was a response to heightened predator pressure (Strum, in preparation), but the fusion process took many years as the baboons renegotiated most aspects of their social life (female and male dominance hierarchies, troop movements, friendships, consorting, etc.). During this lengthy process, the outcome was not clear, unlike in the few fusions in the literature where the event happened overnight. Furthermore, the benefits had costs. Each troop compromised in home range size, in foraging priorities, and in reproductive opportunities after fusion. The second fusion happened 7 years later. Once again, the social and ecological context gave clues. This time, fusion appeared to be a way for a small indigenous troop to gain access to limited patches of *O. stricta* at the front of the* Opuntia* invasion and in the core area of a different and large indigenous group. But here too the outcome was uncertain. At one point, the small troop received enough female and male transfers from the larger study group that the fusion stopped. It also baffled me that the two troops (the study group and the small indigenous troop) continued to return to the* Opuntia* patch for over a year despite mobbing by the resident troop. Infant and female mortality skyrocketed compared to previous years and yet the baboons returned for more mobbing and displacement. The baboons violated the rules of baboon behavior that I had pieced together over the past decades. Eventually the two troops did merge but they did not benefit from access to the cactus until *O. stricta* invaded the study group’s traditional home range. Then the troop returned and the mobbing stopped. The fusion process took over 2 years, like the earlier fusion. However, the newly merged group did not benefit from eating the cactus fruit until 8 years later (Strum et al. in press). I had to wonder whether baboons can make sacrifices in the present for future gain and plan 8 years in advance.

## Invasion of a non-indigenous plant: *O. stricta* (2005-present)

Although I never intended to study the invasion of an exotic plant, *O. stricta* had such an impact on baboon diet, ranging, condition, and reproduction, that I added it to our regular ecological monitoring in 2005. At the start, not being an ecologist, I did not think in terms of an “invasion” and treated the plant as if it were a baboon, recording its ecological preferences, reproduction, and the impact of baboons and elephants on it, not just its impact on the baboons. The continuous ecological monitoring since translocation allowed me to understand the role of different factors in the plant’s spread, including Maasai settlement and the continuous grazing of their cattle, baboons, elephants, topography, altitude, and the plant’s expansion from the point of origin. While I was just guessing about important factors, it turned out that in 100 years of research on *Opuntia* spp., this was the first documentation of its invasion process. Other researchers had made inferences based on historical reconstruction or experiments (Strum et al. 2015).

**Fig. 8:**
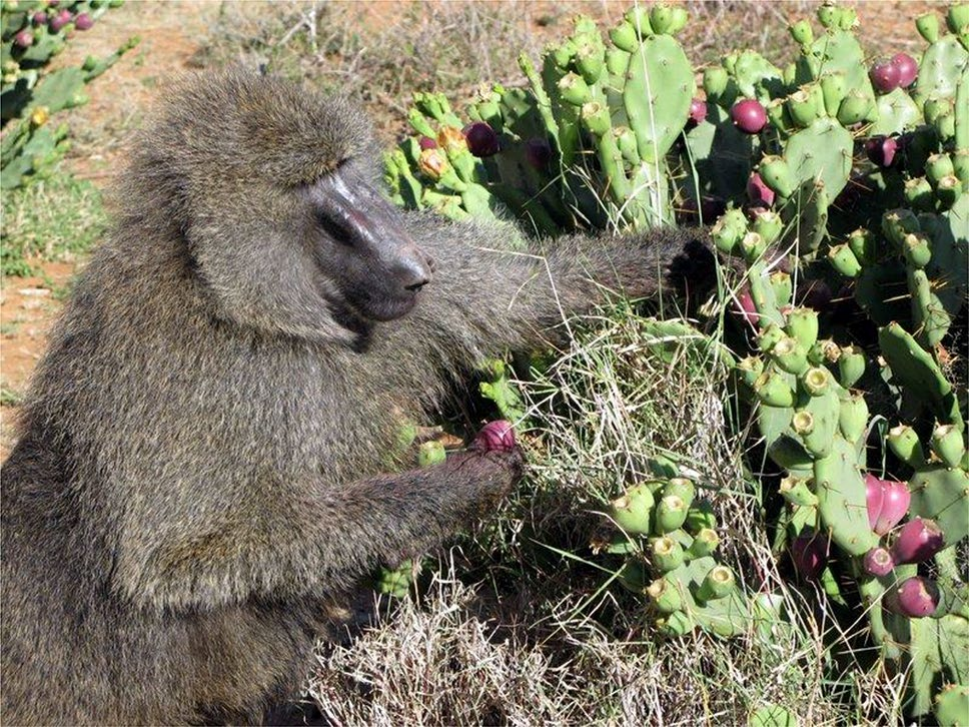
Invasive cactus *Opuntia stricta*

The *Opuntia* story fits in well with my growing interest in “process.” For example, at the beginning of the invasion, baboons dispersed the plant only 100 m from their rocky sleeping sites, and just the sleeping sites closest to the point of origin had any *Opuntia* plants, while those further west had none. Settlements of local Maasai pastoralists (*bomas*) showed a 25-m cordon sanitaire without plants surrounding the* boma*, but the impact of humans started there and peaked 200 m away from the* boma*. Elephants were also significant dispersal agents. They carried the plant further than any other disperser, from the point of origin westward for 5 km along an elephant corridor. After 5 km no cactus plants were present. I called *O. stricta*’*s* invasion a perfect storm because the process of invasion was complex and multidimensional. Continuous heavy grazing by livestock appeared to be the tipping point, a conclusion reinforced by the historical timing of *O. stricta* invasions in the Caribbean and around the Mediterranean. Kenya’s special contribution to the plant’s distribution was its double rainfall regime, which enhanced fruit production compared to that in *O. stricta*’*s* native habitat, the rocky coastal shores of the southeastern United States that has a single period of rainfall each year. Large mammal dispersers in the baboons’ home range replaced the extinct ones in *O. stricta*’*s* native habitat. More rainfall and a greater variety of dispersal agents increased the rate of invasion (Fig. 8)

.

## Lessons learned from watching baboons

I do not have the space to enumerate all the lessons that I have learned from watching baboons. Instead, I will list some of the most important. The comparative method—a basic approach in anthropology and evolutionary studies—is a powerful way to create robust interpretations, even in the short term, as Darwin demonstrated. But long-term studies yield more authoritative accounts and insights about how animals respond to changing conditions over time, particularly in extreme times.

Washburn suggested that I remove all the males at the end of my first study. Fortunately, I did not need to. What Washburn and I never imagined was that my research could benefit from natural and unnatural experiments, nor what these would tell me. The first of these findings was the development of baboon hunting, which I thought would become a tradition because of its many advantages. Then came crop-raiding, followed by translocation. Finally, the invasion of a foreign prickly pear cactus taught me how natural and unnatural processes interact, since humans brought the plant to the area and 50 years later created conditions conducive for its spread. These findings illustrated that what matters to baboons is not always what evolutionary theory predicts.

The baboons constantly challenged me to rethink many commonly held assumptions in the fields of primate science and animal behavior. I even pondered the meaning of reproductive success. Beginning in 1975, sociobiology and later behavioral ecology elevated reproductive success as key to natural selection in primates and other animals. But what time frame should we use for this? Initially, consortship and infant survival were the behavioral surrogates. Then genetic measures of paternity replaced behaviors. But how many generations should be followed and how? Peggy, the most dominant female in the Pumphouse Gang in 1972, whose matriline continued to be top ranking for several decades, has no living descendants that we know of now. Of course, we do not know what happened to her sons, grandsons, and great-grandsons, but that is the point. We need to decide what time scale to use for evolutionary principles like reproductive success and how to measure them. Weiss and Buchanan (2009) and others try to fill the gap between proximate and evolutionary causation. Their framework fits the evidence from the study of baboons better than traditional approaches since these baboons illustrated how evolution is more tolerant than generally assumed. I will leave the questions of how to link proximate to ultimate causes and proximate time to evolutionary time to others.

I was trained as an ethologist in the 1960s so accepted the received wisdom of the times that anecdotes should be excluded from “data.” And yet, despite my training, I also recorded the social and ecological context of behaviors. It has taken me most of my career to realize that these baboon anecdotes were actually systematic and comparative “natural history,” i.e., what Darwin had used so effectively. Now I am unabashed about the importance of returning natural history to primate science to make our interpretations of behavior robust (Strum 2019). Hypothesis-testing science is critical but reductionistic. Natural history provides a way to put back together the complexity we know exists. Thought of in this way, rare events should not be dismissed because they cannot be treated by statistical analysis. Rare ecological and social events matter to baboons (and to humans) because contingency is now recognized as a major factor in evolution, at least by ecologists (Sagarin and Pauchard 2012). Without information on a nighttime encounter one troop had with elephants, I could only describe their home range shift but not explain it. Natural history adds to quantitative studies but does not replace them (Strum 2019).

## Detours from my idealized professional career (1972-present)

Scientific papers make research seem neat and tidy, but it is not. My career also was not neat and tidy. Many unexpected detours added to my perspective on baboons, evolution, science, nature, conservation, and what it means to be a woman professional.

My first detour was writing my thesis on baboon predation and not on the baboon model I had spent so much time studying. Soon I diverged into science studies. Being Washburn’s student meant that the manner in which we do science was always in the back of my mind. Science studies taught me the difference between science with a capital S, idealized science, which valorizes isolated individuals, and science with a small s, the process of research which connects people, history, and machines into networks of actors. I also understood why the early baboon studies only identified large males—at the time, the rest of the group was assumed to be unimportant. Fortunately, the second Wenner-Gren International Symposium, which took place 20 years after the first one, in 1976 (Baboon models and muddles) was on “How and why ideas about primate societies have changed during the relatively brief history of the field of primate studies” (Strum and Fedigan 2000b). In the interim, attitudes changed from primate watchers who did not want to be watched to interesting discussions between primate scientists and those who studied science. Washburn often said that today’s science is tomorrow’s superstition because “facts” are simply the best fit between current methods and reality. As methods change, so do the facts. Now I knew why.

Crop-raiding changed my focus and increased my anxiety. No one had ever watched a naïve group of monkeys become crop-raiders. I needed to test old and new methods and find effective solutions. The raiding led to the baboon translocation, a scientific experiment without a precedent. I could finally relax once the baboons survived and slowly thrived, then took another detour into becoming an invasion ecologist to understand the incursion of *O. stricta.*

Initially, I was slow to publish because I hoped that my original data would come out of the Berkeley computer. However, the speed of publication, which is so crucial today, was not such an issue then. I continued my baboon research and constantly revised my ideas about baboons long before I published my results. I did not publish much from 1984 to 1987 because I was pregnant twice during that period, then from 1989 until 2006 I had to deal with a collapsing back. Miraculous surgery restored some of my function, but from 2006 to 2016 I had to work hard to establish what I could do with my new back. During the long periods of medical issues, I devoted my limited time and energy to keeping the research project going and to my teaching. Slow research and few publications would be characteristic of my whole career because of these many detours, and because I prioritized action on the ground over publishing.

I also devoted efforts to informing the public about baboons, publishing in popular magazines (“*National Geographic*,” “*Wildlife News*,” “*Swara*,” “*Kenya Past and Present*,” “*Animal Kingdom*,” and others) and making nature documentaries (“*Shirley Strum and the Pumphouse Gang*,” “*The Nature of Things*,” “*Moving Day for the Pumphouse Gang*,” and 22 others). Conservation was a detour I had not expected. My conservation journey wasn't easy, but it was necessary. The Uaso Ngiro Baboon Project’s (UNBP) motto became: “Research to understand our present; conservation to guarantee our future.”

Being a woman and later a mother were also diversions from the ideal professional path. As an undergraduate at Berkeley, I searched for women mentors who had both a career and a family, but only found women who had to choose between the two. Fortunately, times have changed with respect to that as well, but equity in science, even in the social sciences, is still elusive. Now, at the end of my career, I am glad that I had both a career and a family. I value my contributions to understanding baboons, evolution, science, and conservation, but as a primate, family and friends really matter.

## From unorthodox to pioneering

The baboons led me to see exceptional things. I now realize that I was pioneering new ideas about baboons and baboon agency, about evolution and science, and about conservation. This began when I recognized that baboon society is socially complex and that female and male dominance hierarchies are not the whole story. Furthermore, non-aggressive social strategies of competition and defense could not happen without a mind. Certainly, baboons have navigational intelligence (Strum et al. 1997) and rely on distributed cognition (Strum and Forster 2001). They also have options, make choices, and are not perfectly adapted evolutionary machines (Strum 2012). Many interpretations I proposed were dismissed at first but are now generally accepted. In fact, it is hard to recognize, from today’s perspective, what all the fuss is about. I hope some of my new conclusions about baboons may yet prove to be correct because they have far-reaching implications. For example, baboons have shown me that evolution is more tolerant than we assume (Weiss and Buchanan 2009), and that baboons make mistakes in real life. Baboons illustrated that the female hierarchy may not be about rank and reproductive success but instead a mechanism for predictability of interactions allowing female-based families, which comprise the majority of a baboon troop, to survive (Strum 2012). As you can see, the baboons have led me down paths I never intended to take, but I have enjoyed the journey.

## Research and conservation: working with communities—respect, trust, commitment, and capacity building (1981–present)

Today, young primate scientists are fully aware of the importance of conservation and of working with human communities around their research sites. That was not the case when I started my career. The few field scientists at that time adhered to the academic ivory tower of “natural” pristine populations and “pure” research, so that even my original study was suspect because it took place on a cattle ranch. My attitudes changed slowly as the baboons encountered conservation threats including habitat loss, fragmentation and degradation due to human population expansion, and invasive species. If baboons did not distinguish the source of changes in the environment (Strum and Western 1982), there was no reason to abandon the study of animals who encountered people, so I continued my work.

**Fig. 9:**
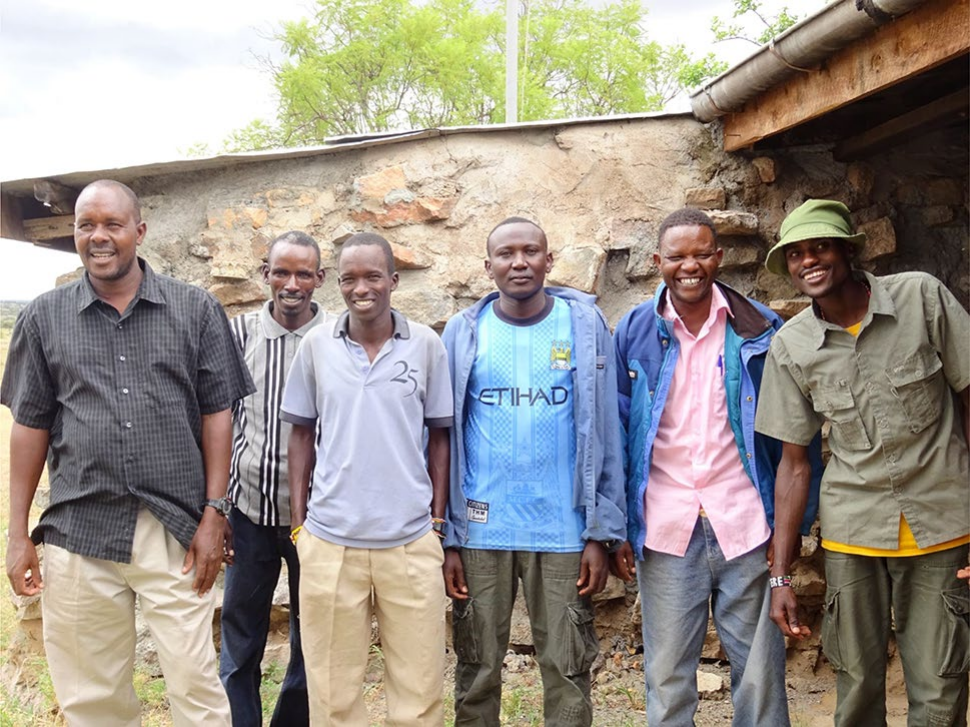
Employing local Kenyans embedded the baboon research in the community

At the start, I defended the baboons against the newly settled farmers who complained about the crops lost to the animals. When I got to know the farmers, our relationships created bonds of understanding. I began to understand how Kenyan population growth put new pressures on people and on land, and how people, wildlife, agriculture, as well as open rangelands, interact. Now I try to consider the human side even when trees are cut for charcoal and sand is harvested from the dry rivers for distant urban construction. I also benefited from my husband’s innovations (Western and Pearl 1989; Western et al. 1994) in community-based conservation. My community-based conservation activities began by using local Kenyans as research assistants in 1981, and the UNBP has relied on them since the baboon translocation for behavioral and demographic baboon data, and for ecological monitoring (Fig. 9)

.

**Fig. 10:**
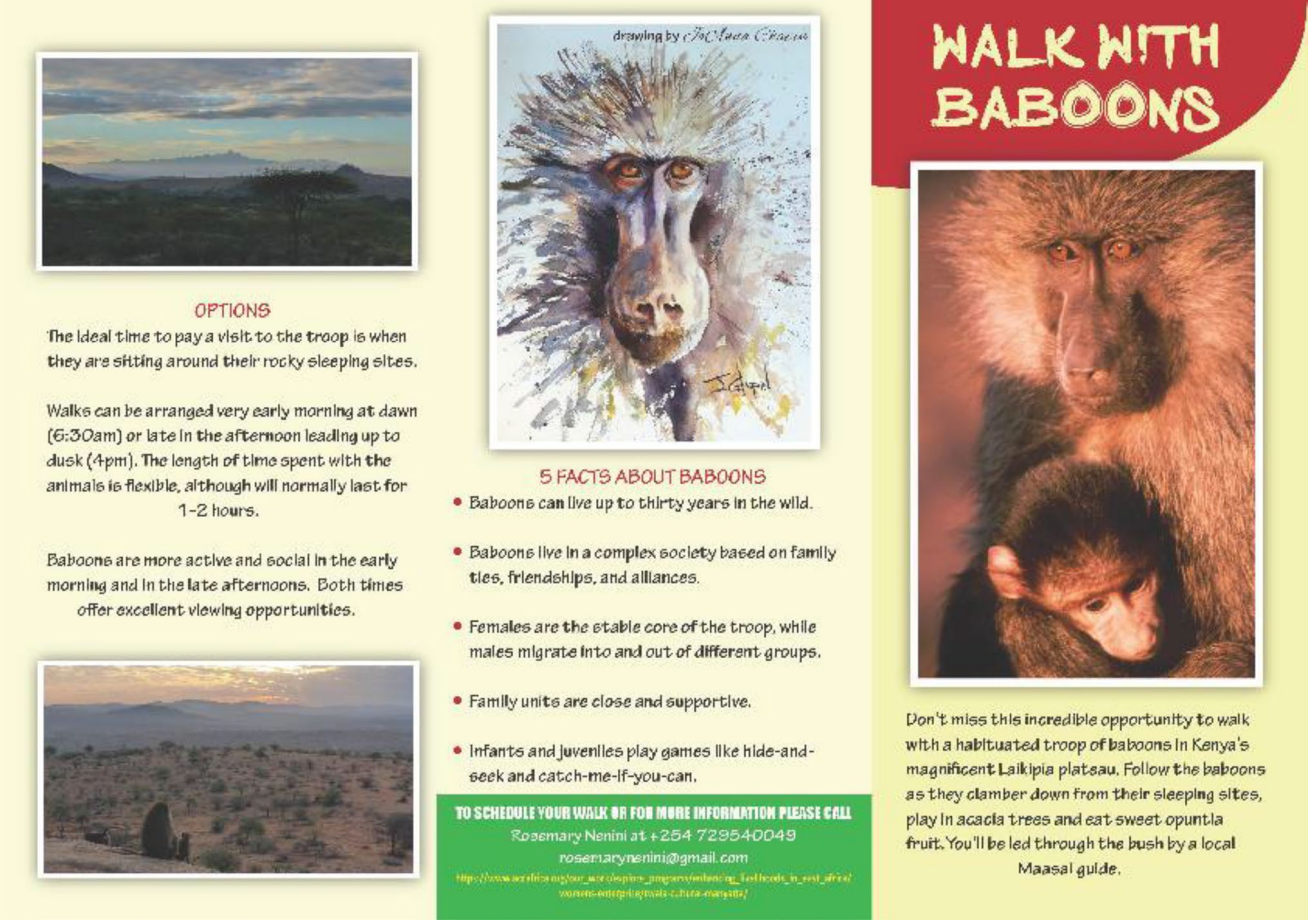
One of the eco-walks developed to generate income for Twala women

Employing local Kenyans has had a knock-on effect as they influence attitudes about baboons among their family and friends. This is a good first step. However, to make a real difference, communities need options, including innovative ways to earn money. During the crop-raiding period, we initiated the Woolcraft Project for the manufacture of carpets from wool that was spun, dyed, and woven by a group of local women and men. Their income offset the crops lost to baboons. After baboon translocation in 1984, we worked with Maasai pastoralists and not Kikuyu farmers, so I had to shift my cultural framework. In 1996, local Maasai women came to me to discuss their idea to preserve Maasai traditions by developing a traditional* manyatta*. Soon they also wanted to earn money, but this “cultural village” was not on a tourist route. UNBP developed eco-walks: walking with baboons (a tourist troop), walking with livestock, and a medicinal plant walk. These brought both local and international visitors to the Twala Tenebo Women’s Cultural Village. Twala women added other activities that created new ways to generate income. For the first time, women had money of their own from employment, ecotourism, the sale of honey, and the sale of aloes for soaps and cosmetics. While most of the income went to members as dividends, a portion also helped elderly and vulnerable members of the community and went into bursaries for girls’ education. Growing Twala enterprises involved training and capacity building. It also meant being alert to emerging problems that needed solving. While the baboons did not have crops to raid after their translocation, they then took young livestock at the end of dry seasons and in droughts: another problem to solve. This is why conservation is never finished, irrespective of whether it is initially a success or a failure (Fig. 10)

.

Education is central to awareness and capacity building, but it is time-consuming and slow. The UNBP has been involved in fostering education since 1981, when we helped build the first primary school in the area. After the baboon translocation in 1984, UNBP expanded its support to several primary schools. When primary education became “free,” the community requested support for newly created nursery schools. When one of the translocated troops moved eastward, the number of nursery schools in the baboons’ home ranges increased dramatically. Partners like the Liz Claiborne/Art Ortenberg Foundation, the African Conservation Centre, the Dutch government, the Rotary Club, US and Kenya, and private donors have contributed money to UNBP’s community work. What UNBP gives in exchange is being on the ground, watching and understanding events, and asking the community what they think is needed. In this system even small amounts of money are effective (Strum and Nightingale 2014).

Field primatologists today have a new respect and concern for their study animals and, increasingly, also for the people who live with these animals. If people are part of the problem then people have to be part of the solution. This is a tenet of community-based conservation (Western and Pearl 1989). UNBP continues to use the best science to create new and better baboon conservation methods that include the participation of local people.

## Lessons from the human age: the Anthropocene

Today is the human age, the Anthropocene. Watching baboons helped me preview what this meant, and led to a new question: “What is nature in this increasingly human-constructed world?” Baboons illustrated how natural selection is being replaced by human selection as the main factor determining the future of species. Baboons, along with crows, coyotes and other smart creatures, are most successful in navigating this new world of the human age. Our task as primate scientists requires reconfiguring ideas of nature to meet the challenges of the Anthropocene, whether in studying or conserving primate species, or in our own lives.

My own small baboon/human piece of the Anthropocene story has shown me that information is important in raising awareness and in education, but I have also learned that success depends on appropriate cultural translations. The Maasai I work with do not divide the world into nature and culture. The term “biodiversity” has no meaning for them. But when I talk about* erematere*, a Maasai term for the connection between the land, the cow, and the family, they understand biodiversity in a way that most of us have only recently discovered. Awareness and understanding are good starting points, but there is one more important step. People seldom change their behavior without alternatives, new options. This is even more true for those living at the edge of subsistence. Together, we need to think outside the box to create tailor-made solutions to problems so that it is possible for human and nonhuman primates to coexist (Strum and Nightingale 2014).

## Things have changed

When I got back from my first field study in 1974, the Berkeley computer took up eight floors and had less computing power than the earliest portable computers. So much has changed. Before, long-term studies evolved organically, but today new projects start with a long-term horizon. Today, teams of specialists are needed as befits the current state of knowledge, while lone rangers like me are dinosaurs. Field experiments are more common but not as frequent as the early ethologists or Washburn had hoped. Breakthroughs in methods include non-invasive techniques that can examine how socioecology impacts individual physiology. There are genetic studies that can identify the father of an infant, replacing previous behavioral surrogates for reproductive success.

Social media has a disproportionate impact, both in democratizing information and as a means of spreading misinformation. A recent paper on elephant conservation and Twitter (Hammond et al. 2022) demonstrates that Twitter posts seldom mention key threats to elephants. Instead, elephant welfare issues like tourist elephant rides are the most frequently discussed topic. Perhaps, as Hammond et al. (2022) suggest, the reason for this is that local stakeholders are not represented on Twitter, so that those who live with elephants do not have a voice. Since social media guides public attention, perceptions and even actions may be misdirected. I have seen a major shift from early in my career in opinions about baboons. Baboons have always had a bad reputation but today it is even worse. I credit this to the impact of the media. For example, “*National Geographic*” made a film about baboon males who steal human infants (which is untrue), while most online videos of baboons are about the serious conflict between baboons and people in Cape Town, South Africa. This conflict became a baboon tradition of eating human food because animal welfare groups captured the media and used the press to control the management methods that were used. A well-known science journalist even argued that she would rid the world of all baboons if she could (Marris 2021). I hope to change commonly held beliefs among scientists and the public using evidence about baboons. Although this is not easy, it is worth trying.

**Fig. 11:**
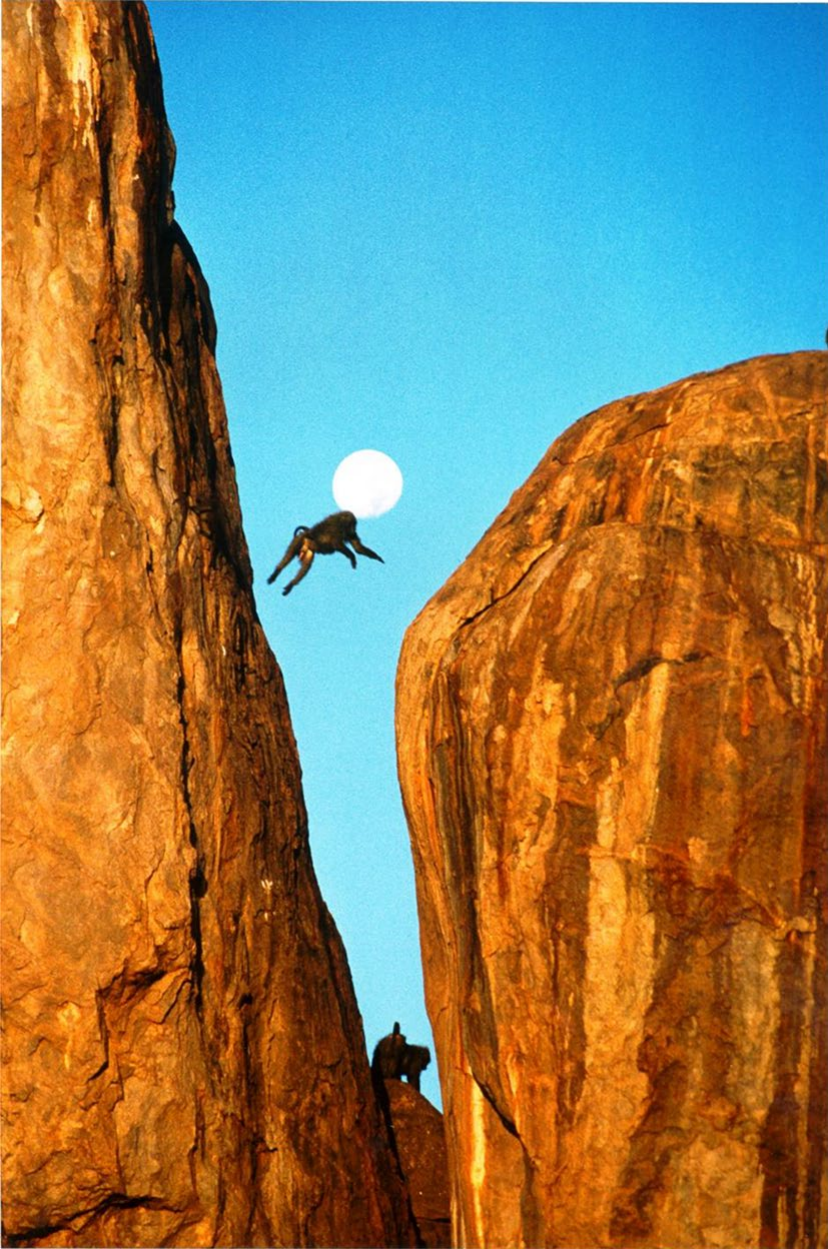
The baboons continue to offer surprises 50 years on

Today, I am grateful that I was part of the early stage of primate studies when each new discovery had a major impact. I am also grateful to be studying olive baboons because, even after 50 years, they continue to fascinate (Fig. 11)

.

## Data Availability

There are no data used in this review aside from published papers. No grants required data depository.

## References

[CR1] DeVore I, Hall KRL, DeVore I (1965). Baboon ecology. Primate behavior: field studies of monkeys and apes.

[CR2] DeVore I, Washburn SL, Howell FC, Bourlière F (1963). Baboon ecology and human evolution. African ecology and human evolution.

[CR3] Forthman-Quick DL, Taub DM, King FA (1986). Controlling primate pests: the feasibility of conditioned taste aversion. Current perspectives in primate social dynamics.

[CR4] Gartlan JS (1968). Structure and function in primate society. Folia Primatol.

[CR5] Gartlan JS (1975) Adaptive aspects of social structure in* Erythrocebus patas*. In: Kondo S, Kawai MA, Ehara A, Kawamura S (eds) Proceedings of the Fifth Congress of the International Primatological Society, 1975. Japan Science Press, Tokyo, pp 161–171

[CR6] Griffin DR (1976). The question of animal awareness: evolutionary continuity of mental experience.

[CR7] Hall KRL, DeVore I (1965). Baboon social behavior. Primate behavior: field studies of monkeys and apes.

[CR8] Hammond NL, Dickman A, Biggs D (2022). Examining attention given to threats to elephant conservation on social media. Conserv Sci Pract.

[CR9] Hausfater G (1975). Dominance and reproduction in baboons (*Papio cynocephalus*). Contrib Primatol.

[CR10] Huxley J (1974). Evolution: the modern synthesis.

[CR11] Langlitz N, Strum SC (2017). Baboons and the origins of actor-network theory. BioSocieties.

[CR12] Latour B, Strum SC (1986). Human social origins: oh please, tell us another story. J Soc Biol Struct.

[CR13] Latour B, Woolgar S (1979). Laboratory life: the construction of scientific facts.

[CR14] Marris E (2021). Wild souls: freedom and flourishing in the non-human world.

[CR15] Mayr E (1942). Systematics and the origin of species.

[CR16] Ransom TW (1981). Beach troop of the Gombe.

[CR17] Rowell TE (1966). Forest-living baboons in Uganda. J Zool.

[CR18] Sade D, Altmann SA (1967). Determinants of dominance in a group of free-ranging rhesus monkeys. Social communication among primates.

[CR19] Sagarin R, Pauchard A (2012). Observation and ecology: broadening the scope of science to understand a complex world.

[CR20] Schaller GB, Lowther GR (1969). The relevance of carnivore behavior to the study of early hominids. Southwest J Anthropol.

[CR21] Smuts BB (1985). Sex and friendship in baboons.

[CR22] Struhsaker TT (1969). Correlates of ecology and social organization among African cercopithecines. Folia Primatol.

[CR23] Strum SC (1975). Primate predation: interim report on the development of a tradition in a troop of olive baboons. Science (80-).

[CR24] Strum SC (1975). Life with the Pumphouse Gang: new insights into baboon behavior. Natl Geogr Mag.

[CR25] Strum SC (1979) Social strategies and the evolutionary significance of social relationships. Circulated manuscript

[CR26] Strum SC, Teleki G, Harding RSO (1981). Processes and products of change: baboon predatory behavior at Gilgil, Kenya. Omnivorous primates: gathering and hunting in human evolution.

[CR27] Strum SC (1982). Agonistic dominance in male baboons: an alternative view. Int J Primatol.

[CR28] Strum SC (1983). Use of females by male olive baboons (*Papio anubis*). Am J Primatol.

[CR29] Strum SC, Taub D (1984). Why males use infants. Primate paternalism.

[CR30] Strum SC (1987/2001) Almost human: a journey into the world of baboons. University of Chicago Press, Chicago

[CR31] Strum SC (1989). Longitudinal data on patterns of consorting among male olive baboons. Am J Phys Anthropol.

[CR32] Strum SC (1991). Weight and age in wild olive baboons. Am J Primatol.

[CR33] Strum SC (1994). Reconciling aggression and social manipulation as means of competition. 1. Life-history perspective. Int J Primatol.

[CR34] Strum SC (1994). Prospects for management of primate pests. Rev Ecol Terre Vie.

[CR35] Strum SC (2002). Translocation of three wild troops of baboons in Kenya. Reintroduction News.

[CR36] Strum SC (2005). Measuring success in primate translocation: a baboon case study. Am J Primatol.

[CR37] Strum SC (2010). The development of primate raiding: implications for management and conservation. Int J Primatol.

[CR38] Strum SC (2012). Darwin’s monkey: why baboons can’t become human. Am J Phys Anthropol.

[CR39] Strum SC (2019). Why natural history is important to (primate) science: a baboon case study. Int J Primatol.

[CR40] Strum SC, Fedigan LM, Strum SC, Fedigan LM (2000). Changing views of primate society: a situated North American view. Primate encounters: models of science, gender, and society.

[CR41] Strum SC, Fedigan LM (2000). Primate encounters: models of science, gender, and society.

[CR42] Strum SC, Forster D, Nowell A (2001). Nonmaterial artifacts: a distributed approach to mind. The mind’s eye: multidisciplinary approaches to the evolution of human cognition.

[CR43] Strum SC, Latour B (1987). Redefining the social link: from baboons to humans. Soc Sci Inf.

[CR44] Strum SC, Nightingale DLM, Russon AE, Wallis J (2014). Baboon ecotourism in the larger context. Primate tourism: a tool for conservation.

[CR45] Strum SC, Western JD (1982). Variations in fecundity with age and environment in olive baboons (*Papio anubis*). Am J Primatol.

[CR46] Strum SC, Forster D, Hutchins E, Whiten A, Byrne R (1997). Why Machiavellian intelligence may not be Machiavellian. Machiavellian intelligence II. Extensions and evaluations.

[CR47] Strum SC, Stirling G, Mutunga SK (2015). The perfect storm: land use change promotes *Opuntia stricta*’s invasion of pastoral rangelands in Kenya. J Arid Environ.

[CR48] Strum SC, Muiruri D, Mburu C (in press) Come be with us: troop fusion as a resource strategy. In: Wallis J (ed) Baboons. Cambridge University Press, Cambridge, UK

[CR49] Sugiyama Y (1965). On the social change of Hanuman langurs (*Presbytis entellus*) in their natural condition. Primates.

[CR50] Teleki G (1973). The predatory behavior of wild chimpanzees.

[CR51] Washburn SL (1951). The analysis of primate evolution with particular reference to the origin of man. Cold Spring Harbor symposia on quantitative biology.

[CR52] Washburn SL (1951). The new physical anthropology. Trans N Y Acad Sci.

[CR53] Washburn SL, DeVore I, Washburn SL (1961). Social behavior of baboons and early man. Social life of early man.

[CR54] Weiss KM, Buchanan AV (2009). The mermaid’s tale: four billion years of cooperation in the making of living things.

[CR55] Western D, Pearl MC (1989). Conservation for the twenty-first century.

[CR56] Western JD, Strum SC (1983). Sex, kinship, and the evolution of social manipulation. Ethol Sociobiol.

[CR57] Western D, Wright M, Strum SC (1994). Natural connections: perspectives in community-based conservation.

